# (Mis-)belonging to the climate-resilient city: Making place in multi-risk communities of racialized urban America

**DOI:** 10.1080/07352166.2022.2160339

**Published:** 2023-02-21

**Authors:** Galia Shokry, Isabelle Anguelovski, James J. T. Connolly

**Affiliations:** a Kean University; b Universitat Autònoma de Barcelona; c University of British Columbia

**Keywords:** Climate resilience planning, green infrastructure, belonging, placemaking, gentrification, climate justice, climate coloniality

## Abstract

Through climate adaptation planning cities are transforming places and relations, most recently via green climate resilient infrastructure (GRI). Yet, GRI’s incorporation into existing, racialized infrastructure systems of urban development, regeneration and finance has raised questions about the socio-cultural impacts and justice dimensions of recent directions in climate adaptation planning and urbanism. While critical scholars highlight the exclusion of historically marginalized residents, this paper’s analysis of the impacts of GRI-driven planning for sense of belonging reveals a complex and multi-faceted experience of gentrification and displacement in the racialized, settler colonial city. Drawing on insights from civic actors about their lived experience of green and climate resilient projects in Boston, Massachusetts, we develop a novel understanding of belonging, which entails degrees of (mis)belonging. Our analysis uncovers three pathways by which climate urbanism shapes belonging into various alienated, subordinated, assimilated and emancipated forms, and reveals the kinds of political subjects and socio-cultural relations that emerge from the lived experience of climate adaptation projects. More broadly, this study sheds light on how less visible placemaking practices and alternative modes of addressing socio-climate vulnerability contribute to climate justice and injustice dynamics.

## Introduction

As climate adaptation planning in places as diverse as Philadelphia, Manchester, Barcelona, Lyon, Medellin and Durban increasingly resorts to green climate resilient infrastructure (GRI), new projects and investments are also transforming places and neighborhoods by altering socio-ecological features and relations. With urban areas at risk from more frequent and intense climate effects, GRI, such as climate-proofed parks, street trees, berms and wetlands are often presented as multifunctional and “no regrets” solutions to building climate resilience while addressing cities’ other social, economic, health and environmental goals. In the United States, the promise of such co-benefits has helped green infrastructure for climate resilience receive wide public support and funding from multiple government agencies and private sources. More recently, an influx from President Joseph Biden’s administration provided “once in a lifetime” federal infrastructure grants that drove a new sense of urgency in local communities to implement climate resilience projects before the funding disappears. These new grants form part of the U.S. federal government’s Justice40 Initiative which aims to recognize and account for the environmental and climate injustices faced by low-income and BIPOC communities by delivering 40% of the overall benefits from federal investments in climate and clean energy to those communities. In some states, like Massachusetts, grant programs such as Municipal Vulnerability Preparedness (MVP)^1^ together with state-level environmental justice obligations, further exemplify how cities are encouraged to bring resilience, neighborhood stability, and justice together, implementing projects that can protect and strengthen vulnerable residents’ places and relations.

Nonetheless, recent experiences with GRI’s incorporation into other existing infrastructure systems of urban development, regeneration and finance have raised questions about the long-term social and racialized impacts and justice dimensions of climate adaptation planning and urbanism. In response, critical urban scholars have highlighted the close ties between green infrastructural development and economic growth strategies (Gould & Lewis, 2021; Long & Rice, 2019) which by prioritizing consensual politics and techno-managerial solutions (Finewood et al., 2019; Kaika, 2017) tend to undermine procedural justice for historically marginalized communities. Critical urban scholars also demonstrate that cities often instrumentalize greenwashing and “climate friendly” lifestyle branding to advance neoliberal governance strategies and attract local and global capital and wealth (Garcia-Lamarca et al., 2021; Long & Rice, 2019) especially to centrally disinvested neighborhoods (Dooling, 2009) targeted for regeneration. A growing strand within the nascent literature on climate gentrification highlights the racially unjust outcomes of the resilient city-making process, particularly how the uneven distribution of green resilient infrastructure leads to environmental privilege for white and wealthier residents and residential displacement and exposure to new climate hazards for lower-income residents and communities of color (Anguelovski et al., 2019; Divalli & Perkins, 2020; Gould & Lewis, 2021; Shokry et al., 2020, 2021). Much of the critical literature on climate urbanism, therefore, points to injustices and forms of exclusion and erasure being reproduced through the intertwining of resilience, regeneration and growth interests, including through continuing processes of climate coloniality (Sultana, 2022).

In this context, an essential question emerges: Can green resilient infrastructure *belong* to less affluent and racialized communities, or do they only serve to dispossess them of healthy and secure local urban environments and futures? Recently, scholars have called for expanding the scope of climate urbanism research beyond either/or scenarios of inclusion or exclusion by engaging with ambiguity, multiplicity and possibility (Castán Broto & Robin, 2021; Robin & Castan Broto, 2020). There is important intellectual space within green urban planning to explore the multi-faceted and sometimes contradictory dimensions of the lived experience of climate urbanism and resilient infrastructure in the context of gentrification and displacement. While scholarship on the politics of resilience planning begins to show how climate urbanism works through processes of racialization to reinforce the differential recognition and valuing of human lives (Anderson et al., 2020; Bonds, 2018; Grove, Cox et al., 2020; Smith & Vasudevan, 2017), the ambiguous and overlapping forms of belonging that arise relative to experiencing climate action remain overlooked and unproblematized in most applications. In particular, there is an emerging recognition of the need within green planning to radically repoliticize the concept of belonging by critically engaging with its settler colonial and racial capitalist legacies of property- and self-ownership, amongst others (Barry & Agyeman, 2020; Butler & Athanasiou, 2013), toward activating alternative forms of belonging, social relations and placemaking.

To further develop the notion of belonging within green resilient planning, our paper asks: How is sense of belonging shaped and (re)configured through green climate resilient infrastructure? What do those pathways of belonging mean for urban climate justice? Here, through analysis of qualitative data, we examine the lived experience of GRI-driven climate adaptation planning and placemaking through the lens of an array of social and environmental justice actors in East Boston, a rapidly gentrifying neighborhood facing mounting climate risks and impacts which has historically been a point of entry and settlement for lower-income immigrants and minoritized groups. We argue that within the combined processes of racialized, settler colonial placemaking built on dis/re-possession and urban greening in response to climate risks, a disaggregated belonging gets constructed across multiple groups. In understanding the spectrum of belonging that arises, our study reveals a complex and multi-faceted experience of gentrification and displacement and generates possibilities for more nuanced green resilient urban strategies.

The paper is organized in the following way: The next section examines the interaction between green climate urbanism and sense of belonging through engaging with scholarship on the processes of racialization and the politics of urban resilience planning. We then explain our case study selection and methodology, followed by detailing our empirical findings according to three pathways: exclusion, negotiation and contestation. Before concluding, we discuss our findings through the lens of mis-belonging in the context of re- or dis-possession. Finally, we close the paper with reflections on what our findings and conceptualizations mean for climate justice at the city and broader scales.

### Green climate urbanism and exclusionary urban transformation

Within the broader scholarship on climate urbanism, the growing critical adaptation literature reframes GRI as part of historically produced and racialized infrastructure systems or networks (Anguelovski et al., 2019; Gould & Lewis, 2021; Shokry et al., 2021) associated with urban planning practices of modernization, development and capital accumulation (Silver, 2019). Often an engineered and standardized product (Finewood et al., 2019), the most institutionalized, visible forms of green resilient and/or storm water infrastructure, may be reminiscent of the “modern infrastructural ideal” described by Graham and Marvin (2001), with a universalizing, homogenizing approach to achieving broader social and political objectives (Lawhon et al., 2018). In the face of universalized climate risks, an ethos of eco-modernism can be found in urban development discourses like green growth and the green tech-based economy that hinges on neoliberalizations and new technologies to reconnect resource flows and create the Smart Sustainable Resilient City (Connolly, 2019). In the name of solving the climate crisis, green resilience is utilized to attract investment, regenerate inner cities (Bigger & Millington, 2019; Garcia-Lamarca et al., 2022) and reconsolidate urban systems through new infrastructure while still promoting limitless economic growth.

Recent scholarship on green climate gentrification (Anguelovski et al., 2019; Gould & Lewis, 2021; Shokry et al., 2020, 2021; Taylor & Aalbers, 2022) demonstrates the uneven impacts of climate urbanism—especially through green resilience infrastructure—from privileging gentrifying neighborhoods to physically displacing residents of color to less climate secure and infrastructure-protected areas (Shokry et al., 2020). Climate gentrification via greening illustrates how resilience infrastructure shifts insecurities, without eliminating them, and thereby adds a new layer of risk to already existing sensitivities (i.e., risks from climate impacts, environmental hazards and urban redevelopment; Cole et al., 2021; Shokry et al., 2021). Thus, the redevelopment of long-disinvested neighborhoods of racialized communities into urban utopian resilience-scapes might also veil the spatial reconfiguration of poverty and segregation through appropriation, displacement (Kaika, 2017) and cultural erasure, possibly making green resilience infrastructure a tool of “racial banishment” (Roy, 2017) and settler colonial urbanism (Porter, Hurst et al., 2020). This critical literature on climate urbanism emphasizes the structural drivers of inequality in the Anthropocene and the failure of neoliberal approaches to climate action to protect poor, residents of color, leading to their displacement, exclusion and persistent vulnerability to climate risks.

### Greening and climate resilient placemaking in the racialized, settler city

In the United States, we may understand this unequal process as a hallmark of American racial capitalism which shapes who belongs to the city—and thus who can lay claim to modernity’s promised future of “human” betterment, growth, development and security (Bonds, 2018; Smith & Vasudevan, 2017). By demarcating those racialized subjects who can be excluded from the (fully) “human” and devaluing often racialized spaces as unlivable (Anderson et al., 2020; McKittrick, 2011; Rutland, 2018), racial capitalism drives racialized zoning, abandonment and infrastructural decay. Yet, racial capitalism is also reinforced through the urban renewal and gentrification of disinvested neighborhoods by repurposing them as new, possible spaces of accumulation for privileged classes (Bonds, 2018; Silver, 2019). Many of these sites were abandoned by inner-city white flight to the suburbs, and benefited little from municipal- and private-led greening in recent decades (Connolly & Anguelovski, 2021). Today, the new socio-spatial configuration of racial capitalism and white supremacy operates in ways that closely associate new green infrastructure, increased and consolidated whiteness, and unequal urban growth (Pulido, 2016) with modernization and development paradigms and policies—from regional and community development programs and public works to the War on Poverty and the War on Terror—that have systematically undermined the strength and security of lower income and Black and Brown communities (Brand & Miller, 2020).

As a part of these same paradigms and policies, some have recently argued that resilience planning conceals and ultimately authorizes the racial violence and coloniality of uneven development by pressing for adaptation to the cyclical effects of a racial capitalist system rather than calling for structural change (Bonds, 2018; Sultana, 2022). In her account of racially uneven development in the aftermath of the 2016 Milwaukee uprising, Anne Bonds (2018) argues that critiques of resilience planning must be situated in a context of racial capitalism, or they may also legitimate the ways resilience reproduces racial hierarchies, such as through colorblind practices (Hardy et al., 2017). In response, scholars have called for more inclusive, race-aware planning approaches that explicitly recognize racial inequalities, distinct vulnerabilities and knowledge discrepancies between residents and planners (Anguelovski et al., 2020; Chu et al., 2016; Fitzgibbons & Mitchell, 2021; Shokry et al., 2020, 2021). While such pro-equality (or equity) and anti-racist strides may lead to more just processes and outcomes, Grove, Barnett, and colleagues (2020) demonstrate how race-aware climate urbanism reaches an impasse—even as it increasingly adopts an equity lens (Bulkeley, 2021)—when granting recognition to claims of injustice requires imposing limits on property, accumulation and growth. That is, when key pillars of white privilege seem endangered or when claims for equity are not articulated in capitalist terms, calls for recognition of and action to undo structural racism are met with dismissive, uncomfortable silence.

In this context of racialized urban development and resilience planning, a historic yet continuing legacy of the American unequal urban landscape is the exclusion of racialized residents from urban natures and green spaces (Connolly & Anguelovski, 2021), further reinforcing who and which geographies belong to the green resilient city and stand to benefit from resilient placemaking. Despite cities’ attempts to address historical gaps in funding greenspace in racialized neighborhoods, parks and gardens—many of which are being redeveloped into GRI—are often not experienced as integrating, welcoming or safe spaces due to racist discourses and practices that define for whom nature is or is not (Byrne & Wolch, 2009; Finney, 2014). Furthermore, in gentrifying neighborhoods, hostile, disciplining and always potentially deadly green space uses and practices by white, privileged newcomers have been described as generating cultural and political “emplaced” displacement (Anguelovski & Connolly, 2021; Hyra, 2015; Wynne & Rogers, 2020)—the feeling of physically inhabiting but having no voice or sense of belonging. As Rinaldo Walcott writes, these [gentrifying neighborhoods] are places where Black and other nonwhite people experience *out-of-placeness*—the feeling that they “do not legitimately belong anywhere that large numbers of white people reside, especially in places now assumed to be white homelands” (2021, p. n/a). Even when residents of color physically remain, scholarship on racialized placemaking points to the exclusion and dispossession of longtime residents of the enjoyment of urban (green) spaces, undermining their sense of belonging, and thwarting more emancipatory approaches to creating a life-affirming sense of place in the green resilient city (Anguelovski et al., 2020; Brand & Miller, 2020; McKittrick, 2011). This emphasizes how placemaking is more than just the specific procedures and outcomes of a planning process, but also who actually experiences the freedom to enjoy those spaces and whose knowledges and histories are represented in them (Amin, 2014; Anguelovski et al., 2021; Ranganathan, 2017).

### Problematizing belonging and (dis-)(re-)possession in green and resilient placemaking

The role of belonging within racialized placemaking is essential to urban green planning because it calls attention to the question we posed in the introduction to this paper: Can green resilient infrastructure *belong* to less affluent and racialized communities or must it only serve to *dispossess* them of [belonging to] healthy and secure local urban environments and natures? In this sense, belonging seems intimately bound up with the having or loss of property- and self-ownership (Butler & Athanasiou, 2013; Roy, 2017) considering the foundational dynamics of settler colonialism and racial capitalism, including land theft and enslavement, upon which the American city has been constructed (Dorries et al., 2022). By understanding cities as indigenous places, the British colonial concept of *terra nullius*—a land that belongs to no one and is up for grabs—is seen today in urban transformations (i.e., regeneration, revitalization, renewal and redevelopment) that combine with state- and market-led green infrastructure programs to perpetuate settler colonial practices through racialized processes of human devaluation, ranking, displacement and dispossession (McClintock, 2018)—what Sara Safransky calls “accumulation by green dispossession” (2017). Today, a plantation logic^2^ formulated on Black and Indigenous dispossession and prohibition of tenure (Walcott, 2021), continues to underlie exclusionary decision-making processes, blank slate urbanism (Derickson, 2014) and public green space uses that reinscribe a white sense of ownership and authority over urban placemaking and who belongs.

Recently, critical urban and environmental justice scholars have called for a deeper engagement with the notion of belonging in planning and placemaking, which is typically articulated in relation to political and cultural recognition (Barry & Agyeman, 2020). Intending to achieve greater distributional or procedural equality, recognition and inclusion alone may not go far enough to address the power, resource and knowledge asymmetries embedded in planning processes (Derickson, 2016; Fraser, 2000), nor to prevent the displacement or de facto exclusion of people of color from neighborhoods targeted by new resilience infrastructure (Grove, Barnett et al., 2020). Such top-down practices may indeed reinforce essentialized identities and/or “conventional notions of belonging” that subvert and incorporate aspirations into a multicultural nation-state with a settler identity (Barry & Agyeman, 2020, p. 15; Smith & Vasudevan, 2017). Furthermore, some rights-based frameworks of recognition may give rise to a problematic sense of belonging wherein “struggles against dispossession too easily become struggles for possession” (Porter, 2014; as cited in Roy, 2017) or repossession. In contrast, critical scholars have suggested that a more complex, situated understanding of (mis)recognition as a continuum of types—such as affirmative, indifferent, and hostile—may allow for a more systematic analysis of the relationship between urban policy and identity and processes of marginalization and stigmatization (Yiftachel et al., 2011).

Given the above, we understand climate resilience planning, in the context of the United States, as embedded in a system of racialized capitalism and white supremacy which depends on racialization to naturalize difference as race and differentially value humanity (Bonds, 2018; Derickson, 2014; Grove, Cox et al., 2020; Omi & Winant, 2014). It is also part of a system that emphasizes the socio-cultural value of possession and that certain surplus populations (Pulido, 2016) are unworthy of belonging (or possessing). This process of dehumanization or creating degrees of humanness—human, not-quite-human, and non-human—(Weheliye, 2014) allows planning to instrumentalize racialized Human subjects in others’ future-building, placemaking, climate protected schemes. In doing so, planning results in deciding who may lay claim to a promised future of “human” betterment, growth, development and security. This mode of deciding benefits and protection depends on the racializing logic of displacement and identifying who belongs (Rutland, 2018), which is embedded in colonial practices. Thus, examining “sense of belonging”—or, in other words, who perceives that they count as an equal member of common humanity (see Fraser, 2006)—provides a window into understanding the kinds of political subjects and socio-cultural relations that emerge from climate resilience planning and adaptation projects. Who belongs, who feels they belong, in which ways, and to what extent, relates to possibilities for future self-determination and how humans make, remake and unmake places (Bedoya, 2013), the city, the nation and the world. As technological fixes to the climate crisis become more and more the measure of last resort, the impact of this process on vulnerable populations is only more acute.

We start from the proposal for “the need to radically repoliticize belonging” (Butler & Athanasiou, 2013, p. 159) through an engagement with its settler colonial (Barry & Agyeman, 2020) and racial capitalist legacies. Here we shed light on less visible placemaking practices and alternative forms of addressing socio-climate vulnerability enacted by marginalized groups in more informal ways (Anguelovski et al., 2021; Robin & Castan Broto, 2020) than engineered GRI which research points to exclusion, displacement and dispossession. By disaggregating belonging as a possible spectrum or mosaic of (sometimes concurrent) experiences shaped by GRI-driven planning and addressing sources of vulnerability emerging in part from climate adaptation planning itself (Ranganathan & Bratman, 2019), our research offers an inductive lens of analysis (Grove, Barnett et al., 2020; Grove, Cox et al., 2020) via the perceptions of civic actors as to how this interplay informs and reinvigorates the politics of climate justice.

## Case background and methodology

The North American city of Boston, Massachusetts, and the East Boston neighborhood in particular, is a critical case for understanding the racialization processes and social impacts and ramifications embedded in green adaptation planning. Since its creation in the 19th century as a single land mass out of five small Boston Harbor islands, East Boston has been a key point of entry and settlement for lower-income immigrants and minorities, with Latinx residents being the most numerous today (Shokry & Anguelovski, 2021). Throughout its history the island developed into a mostly green space deprived and environmentally contaminated neighborhood, dominated by industrial and transportation sector activities along Chelsea Creek and Boston Harbor (Douglas et al., 2012). The creation of the ever-expanding Logan International Airport in the 1920s was perhaps the most devastating infrastructural incursion.

Thanks to the efforts of environmental justice activists, starting in the 1960s, the neighborhood began to enjoy a new era in its environmental trajectory starting in the mid-1990s with the creation of 33 acres of green infrastructure by Massport to compensate residents for the airport’s environmental and health impacts. From the mid-2010s onward, a series of new GRI interventions such as elevated berm landscapes, resilient shorelines, and flood-mitigating parks have been planned along the waterfront as part of municipal efforts to respond to climate risks in a neighborhood where estimates warn that half of the land could be flooded during a major storm in the next 50 years (City of Boston 2017). These GRI are a key tool for advancing two comprehensive municipal initiatives responding to the climate emergency: the 2017 East Boston Climate Ready Plan and the 2018 Resilient Boston Harbor Plan.

We drew on a set of 32 semi-structured interviews conducted in July 2018 and October 2019 in East Boston and updated through in-person informal interviews in July 2022. Among these, 15 interviews conducted with organizers and leaders from key civic action groups and community-based organizations have been central to this study. The civic groups we interviewed were engaged in climate action, environmental stewardship, environmental justice and green and/or climate resilient infrastructural planning. The remaining 17 interviews with city planners, developers, elected officials and environmental nonprofit leaders, alongside an additional review of available, relevant policy, planning and nonprofit documents, provided important case study background and context for the actions of neighborhood groups and organizations. We used a thematic and grounded theory approach to organize and analyze this data into categories of belonging, followed by types of climate urbanism practices.

## Results

We present our results in three subsections, each examining one possible pathway—of exclusion, negotiation and/or contestation—through which GRI-driven climate urbanism and resilience planning disaggregates and shapes belonging into complex, overlapping and sometimes contradictory forms of belonging for East Boston's historically marginalized residents. We explored these broad pathways based on the perceptions of local activists and civic associations apropos to climate resilience-building processes and practices in East Boston.

### Exclusion: The first pathway shaping belonging in the green climate resilient city

We understood exclusion—one broad pathway through which climate urbanism shapes belonging—to take place through the formation of alienated and subordinated forms of belonging. We observed these forms of belonging especially where residents have been excluded from GRI benefits or where externally imposed adaptation and risk mitigation was the main mode of climate urbanism in effect.

#### Alienated belonging

Many East Boston interviewees revealed an intensifying sense of alienated belonging imposed by a growing need for protection from climate impacts while affordable adaptation options and assistance have become increasingly inaccessible to socially vulnerable residents. In this form of belonging, people are separated from a communal drive for adaptive capacity because the definitions of environmental risks and/or the modalities used to mitigate those risks have left people feeling as though they need to go it alone (Nightingale, 2017). Alienated belonging therefore derives from processes in which both working-class homeowners and renters are left to identify and protect themselves from social and environmental risks, thereby creating “atomized subjects” solely responsible for their personal and household security.

For East Boston lower-income homeowners, updated FEMA flood maps have placed many in the flood plain and therefore introduced flood insurance as a form of protection, but also as a new cost burden, since “it’s either (a) inaccessible or (b) totally unaffordable for them,” according to a longtime resident and elected representative. For those already living in the old flood zone, the threat of increased storms and flooding coupled with exponentially rising flood insurance fees has meant new financial and family considerations according to a community-based EJ activist—“Should I try to sell? How long can I stick around?” Working-class homeowners tell of exponential increases in their flood insurance costs from $500 to $3,000 in less than 10 years combined with a tripling of property taxes in the context of neighborhood revalorization and gentrification. Without appropriate and adequate social support, homeowners are thus taking individualized approaches to protection from these multiple risks—including retreat—which intensifies an alienated sense of belonging.

For renters from lower-income ranges, the added cost burdens of flood insurance and repairs is often experienced through rent increases, which have come alongside gentrification. “Many of the folks who would truly benefit from [building better resilience] are actually renters, but the owners of the buildings don’t have the appropriate financial incentives to make the investments they should be making to protect their residents,” explains the elected representative. Other interviewees told us that, on the contrary, the lure of rising rents in East Boston incentivizes landlords to unnecessarily evict renters for climate-upgrading, only to remarket homes at higher prices. These actions create an alienated sense of belonging for all involved, as property owners protect themselves from escalating risks and increase profits, rather than considering a more collective approach or public programs that may help both themselves and tenants (Pelling & Manuel-Navarrete, 2011). Immigrant renters in particular may “self-evict,” displacing themselves rather than facing a court battle to avoid a housing record and deportation risks, and thus experience a deep sense of alienation and isolation.

Additionally, top-down and privatized resilience planning has contributed to creating what some call “islands of resilience.” With the waterfront overtaken by luxury housing through developments such as The Eddy (2016) or Clippership Wharf (2019), affordable or public housing built in the low-lying areas and historic working-class homes are especially susceptible to groundwater flooding and remain unprotected, as a local planner and activist told us: “That means that as sea level rise occurs, it hits the waterfront building, but then it really just moves the water off to … nearby homes and residents and businesses.” As a result, these “islands of resilience” have alienated residents from one another, race, class and housing type. High-income residents have developed an alienated sense of belonging from the surrounding district by living in segregated, high-end protected spaces while working-class residents and homes are also alienated from these new resilient infrastructures.

These examples of alienated belonging derive from processes in which residents are expected to buy their way out of displacement and dispossession or consent to exclusion. Alienated belonging also impacts social bonds in the neighborhood by setting community members against each other—tenants versus landlords, wealthier newcomers versus longtime residents and ethnic minorities versus increasingly white residents—by further polarizing residents according to income, length of stay, race, housing type and resiliency grade. Thus, through new power imbalances and segregated resilience, climate injustice is intensified in the community.

#### Subordinated belonging

Among exclusionary drivers, we also identify forms of subordinated belonging emerging from top-down, city and/or developer adaptation agendas that undermine or circumvent local adaptive capacity efforts, increase climate risks, support gentrification and drive residential displacement. This generates a conditional sense of belonging premised on adopting the top-down adaptation agenda. In East Boston, this form of belonging is generated by GRI-driven planning and development that disciplines, undermines and fails to recognize a full sense of belonging of socially vulnerable and historically marginalized residents.

In implementing adaptation, the city and/or developers may invite participation from civic actors but ultimately set terms which disempower participants, through what Cristina Jackson refers to as “chess game politics” (2019). Alternatively, they may entirely omit resident participation, as a longtime local EJ organizer explained: “It’s always, ‘We know what we’re doing, and we will call you if we need you, and for the record, we don’t need you.’” For example, among efforts to find quick solutions to climate impacts like flash flooding, in 2018, the City of Boston created a deployable flood wall at the end of the East Boston Greenway but, according to a grassroots coastal resilience organizer: “They did a dry run last week and didn’t tell us. We just have to constantly remind the city, ‘Let us know. We’re here, we can help you.’”

Political narratives about housing scarcity as a key driver of housing unaffordability further validate subordinated belonging during adaptation. Rather than advocating explicitly for affordable housing to be central to climate resilient development, a logic of overall housing scarcity ultimately justifies inequitable and unsustainable development with new construction of mostly high-end condos, as told to us by a neighborhood association leader: “the mayor has decided that the way out of the housing crisis is to build; I don’t think he realizes that overwhelmingly what’s being built, and by overwhelmingly, I mean like 90%, is luxury.” The priorities of large-scale developers and power elites are therefore presented as necessary to achieve small gains—such as only 10% affordable housing units in new market-priced developments—for low-income residents.

In relation to the broader waterfront planning process and the degree of community engagement, local organizers regret the numerous instances of procedural exclusion: “We have big developers who are coming from outside with a lot of money and saying, ‘I can pay a million dollars for the permits,’” explains one organizer. Development at this pace, with little permitting oversight or strategic planning guidelines has foreclosed any real inclusion in planning processes, or representation of longtime residents’ interests and needs in planning decisions, ultimately undermining their adaptive capacity. Referring to the luxury high rises and restaurants along the newly green and resilient East Boston waterfront, a community EJ activist explained, “We have increased real estate prices so much that people that used to live there have now been cut off from the water and are leaving the neighborhood because they cannot afford to pay the rent anymore.” Another civic leader suggested that “when they threw The Eddy [a new luxury waterfront development] in there, they knew what they were doing right? I think they’re trying to push people out.” Residents’ belonging and attachment to the coastal environment is ultimately subordinated, and part of the process that leads to their eventual displacement from the neighborhood entirely. These subordinated forms of belonging engendered by exclusionary planning agendas combine with alienated forms of belonging emerging through private, uncoordinated efforts to build resilience and result in residents losing connection with the harbor through displacement while being put at greater environmental risk through maladaptation (Anguelovski et al., 2019).

“Outreach” type consultation in adaptation planning (Finney, 2014) also has subordinating effects. Community groups spoke with great suspicion about such municipal practices: “We [the City] will call out the vote for the top three [of these adaptation ideas], right? Then, we’ll suggest whatever ones we really wanted in the first place anyway.” This especially occurs in what Melissa Checker (2020) calls “predetermined development projects” in which unfavorable trade-offs or options are presented that fail to advance, and more likely undermine, local needs and desires. For example, while a community-led survey revealed that residents want active parks spaces, “of the 30 or so acres of park space Massport manages, only about 3 acres are covered with splash pools and play structures,” a local EJ advocate told us. “The remaining acreage is either considered by Massport as visual landscaping amenities or regulated such that there is no ball-playing, bike riding, frisbee-throwing, etc.” Where the neighborhood has gained active play spaces, there were tradeoffs based on costs: “A lot of [LatinX] communities are playing soccer. It’s easy for the city to have artificial turf … but the downside is that we’re losing green space. We’re losing soil,” explained a grassroots coastal resilience organizer. Subordinated belonging therefore means that lower-income residents’ socio-cultural uses and desires, and affective bonds with place and their natural environment, have been devalued both materially and symbolically through planning and developer decision-making that creates and controls racialized uses and bodies, by opting for less costly and sometimes less resilient, even harmful, interventions. There is no sense of ownership of change, as Binet and others put it in reference to the importance for residents to build power and control over neighborhood developments and investments (Binet et al., 2022).

In sum, municipal planning processes and decisions tend to subordinate urban climate justice goals, community-led responses, alternative resilience practices and uses of green spaces to more lucrative development-prioritized visions while renewing historic mistrust in government leadership. In return, this subordinated sense of belonging (as in the preceding analysis of alienated belonging) generated through exclusionary relations with the city and development interests limits civic action to protect the local community. The final effect is to possibly lead to future self-exclusion from politics and a more disciplined response to climate gentrification, displacement and other growing risks.

### Negotiation: The second pathway shaping belonging in the green climate resilient city

Our examination of climate resilience practices revealed negotiation as a second broad pathway through which climate urbanism shapes belonging. This pathway mainly flows through what we identify as forms of assimilated belonging. This kind of belonging emerges as low-income residents of color negotiate with White power structures (the city and private developers) for small wins, and with new gentrifying residents for cultural presence and recognition.

#### Assimilated belonging

In East Boston, the extensive green regeneration of the waterfront through the development of luxury high-rises, expensive restaurants and a new public esplanade called Harborwalk (Anguelovski et al., 2019) seems to conform to the tastes and socio-cultural behaviors of new, wealthier and younger gentrifying residents. After plans laid dormant for years following the economic recession, development was launched in a sudden, rapid and opaque manner in the mid-2010s. Some groups successfully negotiated for a few additional units of affordable housing or improved access to public use of the waterfront, which is actually a requirement of compliance with Massachusetts General Law Chapter 91. The Harborwalk, with a living shoreline and new marine habitat, is one of these wins, but it is physically and financially dominated by the luxury developments (Shokry & Anguelovski, 2021). Assimilated belonging is an outcome of these imbalanced negotiations in which the city and developers were able to leverage the urgency of climate impacts in the aftermath of Hurricane Sandy to push climate resilience projects and waterfront development (see “Governing through Emergency” in Anderson et al., 2020) that have helped to rapidly intensify gentrification in East Boston.

Assimilated belonging also manifests through longtime residents’ feelings toward their new surroundings and a sense of unwelcome and estrangement generated through new green and climate resilient infrastructures that host gentrifiers and outsiders in this process of settler development. “My kids, or my neighbor, who’s Moroccan or African American [should] be able to walk to the Harborwalk and not feel out of place, says an East Boston coastal resilience activist. … It’s like they need to welcome us, but really, it’s our neighborhood.” This sentiment illustrates a dynamic of assimilation and green climate gentrification: on the one hand, the expectation to conform to hegemonic cultural norms—often inscribed in green space plans, designs and norms of use and behavior; while protecting and sometimes invisibilizing aspects of one’s own cultural identity in order to feel safe and “remain relevant in both worlds” (Finney, 2014). “Most people who created this [park] were white people. They created it so it was more of a calm park.” As more families of color settled in the neighborhood, it started to “feel like it was our park” explained an activist for the East Boston LatinX community. With the recent fast-paced, luxury driven gentrification, however, “it’s been couples visiting, and if you go, you have to be careful because it feels like it’s their park … I think that most things they started to build have been thought for people who are now coming, and not for people who already lived here.”

In other words, residents of color feel newly obliged to assimilate into the cultural tastes of wealthier and whiter newcomers and visitors. “We’re back to being the strangers in our neighborhood.” The diversity of the neighborhood, a longtime working-class and immigrant neighborhood—the Ellis Island of New England—was before perceived as a deterrent to these groups, but now, said an activist from the same organization, “developers are attracted by the multiculturalism they can sell to the kinds of people coming in.” Not only does it draw them in, but they are “banking on an identity of diversity that then you eliminate,” through cultural appropriation and dispossession. As a result, residents feel torn about how to adjust and respond to these changes. On the one hand, residents suffering from racism and exclusion may first feel pride in seeing cultural diversity recognized and valued, drawing visitors and new residents that some may even see as contributing to the social mobility of longtime residents; on the other hand, as others have argued (Hyra, 2015; Summers & Howell, 2019), the marketing of “cool,” “creative” and “authentic” cultural diversity quickly becomes a disadvantage, once again, when it collaborates in their eventual displacement. The additional loss generated by such institutional, environmental and everyday racisms may be the erasure of some socio-cultural practices (i.e., outdoor recreational activities such as barbecuing and large family gatherings) while trying to preserve any remaining sense of security and avoid complete erasure from the neighborhood.

Community groups therefore grapple with perceiving private development and large-scale investment as the only available path to achieving their hopes for neighborhood revitalization and protection from climate risks. A local climate resilience activist explained, “the developers come in, they remediate sites and that’s something that has to be acknowledged.” Furthermore, despite recognition that developer-centered climate urbanism worsens inequalities, there is a sense that this is an unavoidable tradeoff for climate protection and neighborhood improvements. According to the same activist, “Luxury condos or apartments certainly aren’t accessible to me. I don’t make a living wage and many people in the neighborhood don’t, so there’s this dichotomy—it’s gentrifying but at the same time some of them are really building to a high building standard.” This is another dynamic of assimilated belonging, one in which conforming to hegemonic standards for modernization and progress is part of acculturating to a seemingly colorblind and disowned mode of development.

On the other hand, efforts by outside green designers and architects to build adaptive capacity to climate risks in East Boston through community engagement have also been interpreted as unaligned with local needs and desires. “[They] provide a series of options, which are limited—not culturally attuned—created by academics and professionals who don’t understand our neighborhood and the level of education here,” said one EJ activist. Despite the diverse backgrounds of climate resilience professionals, their efforts may be perceived as culturally elitist, harboring conscious or unconscious bias. “[They] never got into a street fight … so, when those people come to the neighborhood, there’s a funny question that they sometimes ask them. Where did you play Little League? With locals?” Widening this cultural divide is a common and shared language of technical jargon and procedures (Swyngedouw, 2009) that facilitates cooperation among city managers, developers/architects and environmental nonprofit staff. Perceiving cultural domination (Derickson, 2016; Fraser, 2000), grassroots activists may feel obliged to use outsiders’ language to be understood or obtain a seat at the table rather than being recognized for their knowledge and belonging. There is little hermeneutical justice in this urban greening process—those opportunities for a marginalized community to “make sense of its distinctive and important experiences on a subject and have the discursive or material tools and spaces to reflect on and share them” (Anguelovski et al., 2020).

In some cases, to advance alternative placemaking models and adaptive capacity to climate risks, local organizations make compromises that may translate into a degree of fragmentation between neighborhood activist groups with different perspectives on the prioritization of issues related to climate protection, development, gentrification, and community identity/belonging. The head of one local environmental organization explains: “We don’t fight displacement, but we are very conscious of it; We’re here to build environmental stewardship. So that’s going to require collaboration with all stakeholders, and I think that’s why we are getting a lot of support.” This relates to how Finney (2014) characterizes the “gatekeeper” who struggles to maintain a double-consciousness, understanding, translating and participating in ways that allow them to remain relevant to both their local sphere and the larger institutional world. There is a sense not only that cooperating with the city and developers is necessary to maintain programs for lower-income and minoritized residents but also that gaining the trust and support of power brokers means keeping a distance from anti-gentrification and environmental justice “fights.” Alliances between housing justice groups and justice-driven environmental stewardship groups do exist, but our analysis of assimilated belonging reveals how they may become more fragile and ad-hoc than strategic through cooperative arrangements with outside groups. This relates to a contradiction in the concept of “bridging” (see, Putnam, 2000) such that collaborating with outside groups may undermine internal neighborhood bonds—a dynamic that describes the struggle of double-consciousness in a society structured by racialized power relations (Du Bois, 2018). Therefore, assimilated belonging may signal an increasing fragmentation of social cohesion—not just between racial and ethnic groups but also within them—and then of activism for social and climate justice. This also demonstrates how processes of racialization are challenged when identifying with the privileges of whiteness appears more beneficial, and then reconfigured through GRI-driven planning.

In sum, civic actors’ negotiations for a more equitable protection against risks may lead to an assimilated belonging that means conforming to both the rules of a structurally unequal system and to new wealthier and whiter residents’ cultural norms. Assimilated belonging is therefore characterized in part by a pressure to compromise (and at times erase) existing social and cultural practices and conform to racial capitalist and settler colonial modes of development in exchange for climate protection, access and participation. The negotiation pathway, and its potentials for cooptation, therefore begs the question—are the benefits worth the tradeoffs? Power asymmetries and systematic incorporation help sustain white supremacy through greening (Connolly & Anguelovski, 2021) and resilience practices while undermining potentials for climate justice (Porter, Rickards et al., 2020).

### Contestation: The third pathway shaping belonging in the green climate resilient city

Last, our analysis of the broad pathways through which climate urbanism shapes belonging reveals that in some cases, civic actors leverage contestation to advance an agenda for more liberating green spaces, transformative responses to climate change, and fostering adaptive capacity and local capabilities. In these cases, a greater degree of community control and equal power relations generates an emancipated belonging through efforts to contest and undo asymmetrical power relations and create more secure and permanent control of local resources (Anguelovski et al., 2020).

#### Emancipated belonging

In East Boston efforts to build adaptive capacity and cultivate emancipated belonging are initiated by civic organizations which aim to not only transform climate resilience practices that have alienated, subordinated and subsumed marginalized residents and their identities, but also to (re)create affective bonds with local green spaces and the Chelsea River and Boston Harbor. They organize alternative placemaking and recreational activities that reassert their equal citizenship and belonging in the neighborhood. Those activities also make these spaces more visible while preparing residents for climate impacts through education and developing a familiarity and comfort with the non-human urban environment. For example, renatured shorelines, beachfronts, kayak docks and other recreational infrastructure ideally improve access to blue and green spaces for some minority and immigrant residents who may have a lower sense of comfort and safety next to waterbodies and green spaces (Finney, 2014; Irwin et al., 2011). To address this issue, several nonprofit organizations provide environmental, safety and stewardship educational programs for residents that foster connection with and knowledge about their natural environment. The director of the community-based organization, Harborkeepers, explains “We are teaching people body safety thermals, climbing on to the boat, climbing on to the fire truck … People never thought this could happen in East Boston, kids learning how to make rope, learning how to tie knots, learning about shells.” Through a climate nature program, students also learn about soil, trees and flooding as well as the urban systems that control and exacerbate climate risks: “We had a lesson on storm drains. Whoever talks about storm drains to kids? What happens when the water doesn’t flow? … How do we advocate improvement of these storm drains?” These kinds of programs support knowledge- and confidence-building in residents to protest inequities in risk and protection and advocate for neighborhood improvement, while fostering inclusivity and spatial agency over amenities, landscapes, and assets (Montgomery, 2020): “everybody can use the kayak, regardless of their income. So, it’s an equalizing program,” explains a local EJ activist and civic leader: Eventually, those activities (re)build social ties within the neighborhood: “It puts families who never have that experience, together, and it makes a precious moment for them which improves their life.”

**Figure 1.**
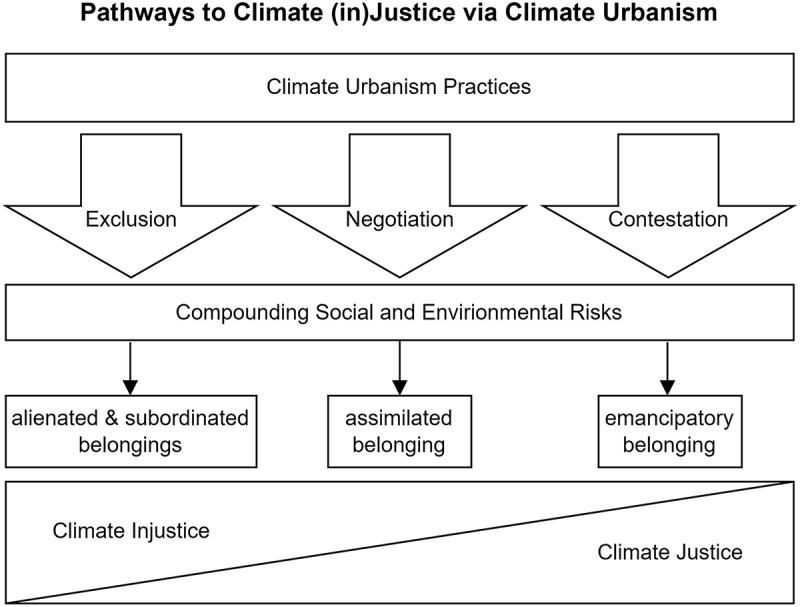
Climate urbanism in East Boston shapes multiple forms of (mis)belonging and climate (in)justice via exclusion, negotiation and contestation.

Emancipated belonging is further created by the activities civic groups organize and foster around historic and recent neighborhood green spaces. Parks built in the 1990s and 2000s to compensate for air, water and noise contamination from industrial activities and the nearby Boston Logan Airport (Douglas et al., 2012), including Piers Park, the East Boston Greenway (Figure 1), Bremen Street Park and Bayswater Street Park, have been enormously popular with locals. For example, the Bremen Street Community Garden, which opened in 2007, has provided Latinx and other families the highly popular pastime of growing fresh vegetables, herbs and flowers and the possibility of enjoying a peaceful, green space in a dense and heavily trafficked neighborhood. Eastie Farm is another community garden started in 2015 by residents who turned an overgrown, underused lot into a space for growing fresh food, accessing urban nature, and building new community ties, while offering educational programs on composting, sustainable growing techniques and environmental stewardship and resilience (Shokry & Anguelovski, 2021). Some programs also attempt to restore residents’ connections to the early maritime history of East Boston and its historic waterfront. Harborkeepers’ director stresses, “We’re asking for this coastal, quote-unquote, community to be allowed to be coastal, which means interacting with the water, which means learning about the environment, which means accessing any place and not feeling like it’s private.” Through these efforts to secure access to green and blue spaces, civic associations have been demanding a restorative and reparative agenda for green resilient infrastructure, and climate urbanism more broadly, wherein neighborhoods that have suffered from historic harms may find reconciliation and recovery through relations of care and repair with urban nature (Anguelovski et al., 2020; Low & Iveson, 2016; Porter, Rickards et al., 2020) and a more emancipated sense of belonging. At the same time, they point out that intensifying green privilege is today limiting such healing and liberation while generating new risks and insecurities. Other nonprofits such as Greenroots are also mobilizing in order to regain ownership and sense of place in the newly greened waterfront by mobilizing Latinx residents around activities such as weekly Green Walks. In those walks, Latinx community leaders share the history of East Boston as a place of immigration, industrialization, contamination, and greening made possible by a white, elitist appropriation of space and resources, a space which they also aim at reclaiming from gentrification through community organizing and critical storytelling for green justice.

Other programs have a more explicitly political and strategic goal of achieving emancipatory belonging by changing power asymmetries in the way climate risks are addressed and engaging future generations to shape the direction of their neighborhood through science-led activism (Martinez-Alier et al., 2011) and other knowledge-centered work. Local youth are leading community-based participatory research and bringing findings to community meetings where climate action alternatives are discussed alongside other issues from “youth violence, the lack of safe places for young people to get together” to immigration and residential displacement, explains the Neighborhood of Affordable Housing (NOAH) climate programs manager. They aim “to educate them and let them know what other people were saying and thinking, then seek their support to move forward on solutions.” More recently, NOAH co-organized a Resiliency Summit in 2022, inviting youth leaders to become “experts and know that they are experts” on climate change, especially on related issues of extreme heat, tree canopy, air quality, food insecurity, flood prevention, digital divide, youth growth, immigration, and housing, and then be direct advocates to the City about how the Green New Deal can be justly operationalized in East Boston.^3^ Youth are thereby leading conversations about the neighborhood’s future and expanding their own and other community members’ knowledge, building adaptive capacity and constructing a stronger platform from which to advocate for the neighborhood. Through this emancipated approach, they are claiming for equalizing and “reciprocal relations” (Finney, 2014) with the city. According to a founder of the ClimateCare initiatives: “[They would] try to meet with the relevant people and say, hey, this is what we see the problem as, here’s what people say about it, and this is the group of people who asked us to come on their behalf and discuss it with you. We’re suggesting this, what do you think?”

In a similar manner, other local groups have taken an emancipatory and socially transformative approach to building political power and addressing multiple risks with climate change, infrastructural challenges, and community-centered economic development included among them. They have, for example, created workshops to train local leadership in how to influence city decisions on issues such as immigrants’ rights, electrical power and land use, launching also a campaign called Right to Remain to better understand and act on the growing threat of displacement in East Boston: “this is the reason they’re involved and interested in solidarity economy whether that expresses itself as control over land or direct control and ownership over businesses,” explains a city activist about Neighbors United for a Better East Boston (NUBE). As an alternative to profit-motivated, capitalistic modes of placemaking, residents of the Latinx community, have organized the Center for Cooperative Development and Solidarity to address various local issues using popular education techniques drawn from cultural knowledges and traditions of their home countries. “They can ignore a person, but they can’t ignore a group of people. And our objective is to gain enough economic and political power to have an impact on decision making in our neighborhood.” They are doing the work of building political power and agency, not just in negotiating neighborhood changes, but actually setting the agenda for which changes should take place while defying socio-cultural erasure. “This empowers people. It makes people feel they belong here. … [t]he best way to stay here, is to make a difference, to make culture here,” explains one of its founders.

In sum, these efforts support an emancipated belonging by contesting the pathways and multiple risks driving alienated, subordinated and assimilated belongings through climate resilience planning and asserting equalizing and reciprocal relations in support of expanded possibilities for life (Montgomery, 2020). These actions affirm the “racial and socioecological indispensability” of residents of color (Pellow, 2016) through everyday acts of resistance—even informal and less visible ones—to risks by strengthening social and environmental bonds. Projects that characterize this pathway may comprise decolonial and abolitionist practices aimed at cultivating a culture of care, equal human dignity and community wealth and economic development through recognition of multiple historically produced vulnerabilities and seeking healing through symbiotic relations with land and urban ecologies (Anguelovski et al., 2021; Heynen & Ybarra, 2021; Ranganathan & Bratman, 2019). Crucially, many of these practices that shape emancipatory belonging fall outside of the realm of the “environment” and “climate” narrowly construed but are vital for building climate justice in that they foster networks of solidarity (Ranganathan & Bratman, 2019).

## Discussion: Belonging, dispossession and justice in the climate resilient city

This paper has explored the placemaking practices of civic actors in relation to (green) climate resilience planning in East Boston to understand the socio-cultural dimensions of the lived experience of green resilient infrastructures, in particular for sense of belonging. We show how belonging in the green climate resilient city becomes disaggregated into many context-specific, complex and overlapping forms through experiences with GRI-driven climate urbanism and placemaking. When understood relative to racialized capitalism, this process of disaggregating belonging is a social force that creates degrees of humanness—human, not-quite-human, and non-human (Weheliye, 2014)—and of who may lay claim to a promised future of “human” betterment, growth, development and security (Anderson et al., 2020). This mode of deciding benefits and protection depends on the racializing logic of identifying who belongs, which is also embedded in colonial practices (Rutland, 2018) of the settler colonial city.

First, by highlighting a spectrum of belonging, we begin to better understand how green climate infrastructure is lived and becomes embedded in residents’ way of life and response to risk (not just climate risks). In the critical urban literature, most examples of (green) resilient infrastructure point to the exclusion, residential displacement and dispossession of socially vulnerable residents by gentrification and neoliberal processes (Gould & Lewis, 2021; Hardy et al., 2017; Shokry et al., 2020; Tubridy, 2020). While we do not refute these findings, our analysis of the impacts of GRI-driven planning and urbanism for sense of belonging, reveals a complex and multi-faceted *experience* of gentrification and displacement in the settler colonial city, that manifests as the kinds of political subjects and socio-cultural relations emerging from climate resilience planning and adaptation projects. These findings connect climate urbanism and climate gentrification scholarship with urban research demonstrating that displacement via gentrification is not only a physical process of removal but also a political and sociocultural one that occurs even when people stay (Hyra, 2015; Summers & Howell, 2019). By focusing on these specific outcomes for sense of belonging—the shaping of alienated, subordinated and assimilated belongings—in a way that has not been done before, our findings further show that dispossession occurs through the loss of the capacity to comfortably use and enjoy public spaces due to senses of misbelonging as much as through physical loss. In illustrating the consequences of GRI for the placemaking capacities of socially vulnerable residents, as told by civic actors who are striving to create a more just and resilient East Boston, our case study highlights the role of climate gentrification and displacement in reconfiguring sociocultural belonging and a sense of ownership.

Second, the paper also builds on scholarship demonstrating the role of settler colonial and white supremacist logics in urban greening (Connolly & Anguelovski, 2021; McClintock, 2018; Safransky, 2017) and resilience practices (Bonds, 2018; Grove, Barnett et al., 2020; Grove, Cox et al., 2020; Ranganathan & Bratman, 2019) by specifically emphasizing how these logics pertain to climate gentrification. Ananya Roy (2017) argues that “racial banishment” may more accurately describe the process of gentrification, better than capital accumulation and displacement because it may be understood as an instantiation of white supremacy through disciplining and cleansing communities of color. Our study grounds this notion in specific processes of environmental and climate (in)justice, which reveal the cruel irony of racial banishment that emerges as residents strive to protect their neighborhood through greening, climate resilience and addressing underlying social vulnerabilities. In Roy’s example, racial banishment results from evictions and housing insecuritization. The East Boston case demonstrates the various disruptions of an intimate process of finding healing, safety and belonging through improving one’s neighborhood and deepening social and ecological bonds as a coastal community. This nonetheless connects racial banishment with green gentrification scholarship that has examined this paradox in depth (Gould & Lewis, 2017). Residents of color are seemingly punished for attempting to make a better life and further building what urban environmental justice communities have fought for since the 1990s throughout the United States, namely comprehensive neighborhood improvement and place reconstruction through environmental efforts (Faber & Kimelberg, 2014). Climate gentrification may therefore be understood as a process emerging from a combination of perceived risks from climate change and from elite-driven, colorblind or perhaps color-averse, climate urbanism strategies that ultimately aim to replace longtime racialized residents.

Thirdly, our paper also responds to calls for analysis of climate urbanism interventions that are not “just neoliberal nor purely radical but that fall somewhere in between” (Robin & Castan Broto, 2020), and outside state- or market-led strategies (MacGregor, 2021). In reflecting on resilience, scholars note the flexibility of the concept and call for attention to its different interpretations by actors, the knowledge structures supported, and the different interests and power dynamics embodied (Cox, 2022; DeVerteuil & Golubchikov, 2016; Tozzi, 2021). Similarly, by using an inductive approach (Grove et al., 2020, 2020) to examining the lived experience of GRI, we highlighted complex and ambiguous interpretations of new green and resilient infrastructure and even surprising ways of knowing, feeling and belonging that represented more caring and emancipatory ways of constructing and greening cities in order to attend to the alternative claims and desires of marginalized groups (Amin, 2014; Anguelovski et al., 2020; Maurer, 2020; Ranganathan & Bratman, 2019). These practices may not be directly confrontational; however, they play a critical role in building place attachments and therefore resistance to displacement. They may also generate bespoke adaptation options and greater political power to advance climate action (Chu et al., 2016) with a restorative and reparative agenda via the recognition of multiple historically produced vulnerabilities (Anguelovski et al., 2020). Our analysis therefore demonstrates that while GRI-driven planning tends to hasten racial banishment and exclusion, there is also evidence of more inclusive and rehumanizing forms of climate action. However, given the context of racially uneven development, settler and green growth machine politics, even these collective efforts toward climate resilience and environmental stewardship do not seem to achieve an entirely emancipatory belonging that may signal a fully abolitionist climate justice (Ranganathan & Bratman, 2019). Residents and leaders still struggle as a part of these programs and others with the assimilating, subordinating and alienating aspects of GRI-driven development. Further research on climate gentrification and urbanism should examine the spectrum of belonging in other urban contexts, especially how people seek to create more emancipatory and fully humanizing forms of belonging which would illuminate possibilities for more just cultural transformations in the context of climate change. This analysis can be deepened through an intersectional approach to climate justice (Amorim-Maia et al., 2022) by exploring belonging across a spectrum of multiple identities.

Therefore, community practices in East Boston reveal how different forms of belonging may help, or hinder, the struggles of marginalized urban neighborhoods and communities in building resilience, owning change, and transcending local politics and even compelling global movements for climate justice. Sense of belonging resonates at various scales from the very local to the national and the global. Connecting the cultural-political aspects of urban displacement, climate coloniality, and racialized dispossession via a dual process of gentrification and climate change adaptation—both of which have planetary drivers—may be key to theorizing climate justice at various scales and strengthening global mobilization efforts for more transformative adaptation (Goh, 2020; Shi & Moser, 2021). Future research could conduct the kind of climate gentrification analysis we have done here—via the racialized forms of belonging emanating from exclusionary urbanism—across cities and at transnational and/or planetary scales (see, Blok, 2020) and to identify the actors, histories and politics behind resilience strategies in those differing contexts (Naef, 2022).

Lastly, through this analysis we primarily tease out the interlock of seemingly contradictory concepts like belonging and (dis)possession, that appear entangled in local place- and infrastructure-making practices. The East Boston case helps elaborate on what Roy (2017) calls “(dis-)possessive collectivism”—a political potentiality related to personhood and property—by illustrating how the perpetual challenge of securing permanent claims to home and land shapes identities and encounter which in turn shape possibilities for climate justice. Climate urbanism practices that subvert alienated, subordinated and assimilated senses of belonging may be key along the pathway to forming decolonial and emancipatory senses of belonging, free from “possessory politics” (Porter, 2014; see, also Roy, 2017) and ”the governmentality of property ownership and self-ownership” (Butler & Athanasiou, 2013, p. 159). Recently, however, a group of banks, insurance companies and corporations donated to the creation of Piers Park III, a world-class climate resilient park budgeted at $40 million, which has also been allocated federal funding. In the construction of the new park and new green infrastructure, preserving the belonging of longtime residents will be even more important as the sense of belonging and ownership of corporate giants may grow increasingly stronger along this and other remaining open spaces of East Boston’s historic coastline.

## Conclusion

This paper contributes to a burgeoning interest in understanding the socio-cultural implications of urban climate action at the neighborhood scale by disaggregating the effects of GRI on residents’ sense of belonging. It does so through the analysis of an assemblage of ordinary, ambivalent, radical, incremental, (in)visible, and/or (in)formal practices responding to everyday risks and larger-scale threats. Our case study of East Boston demonstrates three main pathways through which belonging is shaped by GRI. First, we see how *alienated* and *subordinated* senses of belonging emerge via an exclusionary climate urbanism pathway which constrains visions for alternative futures and social and environmental placemaking models, as well as drives displacement and engenders distrust of climate protection. Second, we also found that in East Boston the pathway of negotiating with development and city powerbrokers led to an *assimilated* sense of belonging, one that means both conforming to the rules of the game in a structurally unequal system and to new wealthier and whiter residents’ cultural norms (i.e., racial capitalist and settler colonial modes of development in order to access climate protection). Third, our analysis also revealed that a contestation pathway could lead to a more *emancipatory* sense of belonging through placemaking and adaptation practices that challenge the pathways and multiple risks driving alienated, subordinated and assimilated belongings. These practices in East Boston include the education, youth power-building, stewardship, and environmental protection work led by civic groups—some more explicit than others about addressing structural drivers of inequality—as well as everyday and informal, less visible acts of resistance to risks through strengthening social and environmental bonds. Sometimes working together in loose alliances, a growing sense of emancipatory belonging can support and sustain climate justice in the community.

The question of for whom is the green resilient city is more than a question of equity, right to the city or even recognition. It is also about the experience of belonging to a common humanity which relates to how people make, remake and unmake places, the city, the nation and the world. Green climate resilient infrastructure participates in the system of relations governing this question and ultimately shaping the cultures and societies produced through these efforts and infrastructures. If, on the other hand, our reading of belonging is opened and expanded, allowing for more than an either/or understanding, we may learn about the complex, ambiguous ways in which people actually belong, which may further shed light on less visible placemaking practices and alternative forms of addressing socio-climate vulnerability.

## Notes

1. Municipal Vulnerability Preparedness. (n.d.). *Environmental Justice and Equity Toolkit*. Resilient MA. https://resilientma.mass.gov/mvp/content.html?toolkit=justice.

2. See authors of the Afropessimist tradition: Sexton, 2010; Sadiya Hartman, 2007; Wilderson, 2010 for “afterlife of slavery” and what Kathrine McKittrick (2011) calls “plantation futures.”

3. For more information see: https://www.youtube.com/watch?v=POWfoyFolI0
